# Wavenumber Imaging of Near-Surface Defects in Rails using Green’s Function Reconstruction of Ultrasonic Diffuse Fields

**DOI:** 10.3390/s19173744

**Published:** 2019-08-29

**Authors:** Hui Zhang, Haiyan Zhang, Jiayan Zhang, Jianquan Liu, Wenfa Zhu, Guopeng Fan, Qi Zhu

**Affiliations:** 1 School Institute for Advanced Communication and Data Science, School of Communication and Information Engineering, Shanghai University, Shanghai 200444, China; 2 School of Urban Railway Transportation, Shanghai University of Engineering Science, Shanghai 201620, China; 3 School of Mechatronic and Automation Engineering, Shanghai University, Shanghai 200444, China

**Keywords:** wavenumber imaging, diffuse field, full matrix, near-surface defects

## Abstract

Wavenumber imaging with Green’s function reconstruction of ultrasonic diffuse fields is used to realize fast imaging of near-surface defects in rails. Ultrasonic phased array has been widely used in industries because of its high sensitivity and strong flexibility. However, the directly measured signal is always complicated by noise caused by physical limitations of the acquisition system. To overcome this problem, the cross-correlations of the diffuse field signals captured by the probe are performed to reconstruct the Green’s function. These reconstructed signals can restore the early time information from the noise. Experiments were conducted on rails with near-surface defects. The results confirm the effectiveness of the cross-correlation method to reconstruct the Green’s function for the detection of near-surface defects. Different kinds of ultrasonic phased array probes were applied to collect experimental data on the surface of the rails. The Green’s function recovery is related to the number of phased array elements and the excitation frequency. In addition, the duration and starting time of the time-windowed diffuse signals were explored in order to achieve high-quality defect images.

## 1. Introduction

In the transportation industry, the railway is exposed to harsh environments all year. The corrosion emerges at welding positions of the rail, which leads to interior hole generation. This not only requires costly maintenance strategies but also brings potential security issues. Therefore, nondestructive testing (NDT) plays an important role in the monitoring of railway safety at present. Ultrasonic nondestructive testing (UNDT) uses controllable mechanical waves to detect internal defects in materials, which is one of the most commonly used methods [1]. Such waves can be generated by piezoceramic transducers [2,3], metamaterials-based sensors [4,5], and so on in order to detect and localize damages. As a new type of data acquisition system of UNDT, ultrasonic phased array is increasingly adopted with simultaneously signal transmitting and receiving, which is mostly made of piezoelectric composite. It offers high inspection efficiency, strong real-time performance, and low cost. However, the nonlinear effect is produced due to the influence of the electromechanical conversion and mechanical vibration, which masks the early arrival signals. Not only the scattering information but also the residual information of the excitation signals (e.g., the ultrasonic reverberation inside the transducer) are included in the received signals. Moreover, the compact-arranged elements produce nonlinear saturation of the electromechanical crosstalk, which makes the received signals more complex. Eventually, these factors cause the formation of a blind zone of several millimeters in engineering materials during ultrasonic inspection. Transducer wedges can be added to reduce these effects, but at the expense of ultrasonic energy loss [6,7] and the appearance of artifacts in the imaging.

In order to better solve the imaging problem of near-surface defects, the method of impulse response reconstruction has become a research hotspot recently. For example, Park [8] computed the correlation between the impact response and the training impulse response functions based on a time-reversal concept. The training impulse response functions are obtained by repeated measurement of various potential impact locations. A more effective approach is to make full use of the diffuse field or ambient noise. This method was first demonstrated by Lobiks and Weaver [9,10] in 2001. The results showed that the direct response between the two sensors can be restored by the cross-correlation of the diffuse field signals, which corresponded to the Green’s function response between them. Based on this, Sabra [11] proved that the coherent guided waves extracted from the diffuse fields were sensitive to the defects in the thin aluminum plates. In seismology, Chaves [12] used the cross-correlation of ambient seismic noise to extract direct seismic response between two points on the earth’s surface. Artman [13] and Campillo [14] pointed out the feasibility of extracting Green’s function extraction from the noise correlation function (NCF) in many fields, including ultrasonic [15,16], underwater acoustics [17,18], and medical imaging [19]. However, application of the Green’s function reconstruction method in the post-processing of ultrasonic full matrix signals is hardly ever reported. A potential application of the use of this method is to address the physical limitations of phased array. 

As a post-processing technique of ultrasonic phased array, the total focusing method (TFM) is a delay-and-sum imaging technique based in the time domain where the area of inspection is discretized into a grid and the signals from every transmit–receive pair are subsequently focused at each pixel and summed [20,21], which is derived from the B-scan. The advantages of a large scanning range and full matrix information extraction make it sensitive to the detection of small defects. However, this algorithm has poor lateral resolution and low imaging efficiency. An accurate imaging technique, the frequency wavenumber algorithm, has been successfully developed for the synthetic aperture radar (SAR) to tackle this problem based on Fourier operation [22,23,24]. This technique correlates the Fourier component of the measured SAR signals with the Fourier component of the imaging target. Originating from the wave equation inversion theory, it is also known as wavefront reconstruction [25,26]. Meanwhile, the basic reconstruction principle is the Fourier decomposition of the Green’s function. According to the wavefront propagation, this algorithm provides an idea for the inverse solution of the target. Compared with TFM, the capability of the wavenumber algorithm in the lateral resolution has also been enhanced [27]. The wavenumber algorithm takes full advantage of the Fourier operation, thus greatly improving the computational efficiency. The Green’s function retrieval theory and wavenumber imaging algorithm are combined to solve the imaging problem of near-surface defects, which is still under development in ultrasonic nondestructive testing.

In this work, near-surface defects in rails were explored by different ultrasonic phased arrays with different parameters. The chapters of this paper are organized as follows. First, the theory for the Green’s function retrieval based on the cross-correlation of diffuse fields is introduced in Section 2. Then, the reconstructed matrix is further weighted combined with the full matrix directly obtained. Meanwhile, the experiments were executed, and results indicate that it allows the near-surface information to be obtained and reduces background noise. In Section 4, the wavenumber algorithm was applied to locate the near-surface defects, which has better performance compared to the conventional B-scan. Subsequently, the influence of different parameters on the reconstruction of the Green’s function is further discussed, which provides operational guidance to the optimal diffusion field signal collection. Finally, conclusions are given in Section 5.

## 2. Green’s Function Retrieval Based on Diffuse Field Full Matrix

**Figure 1:**
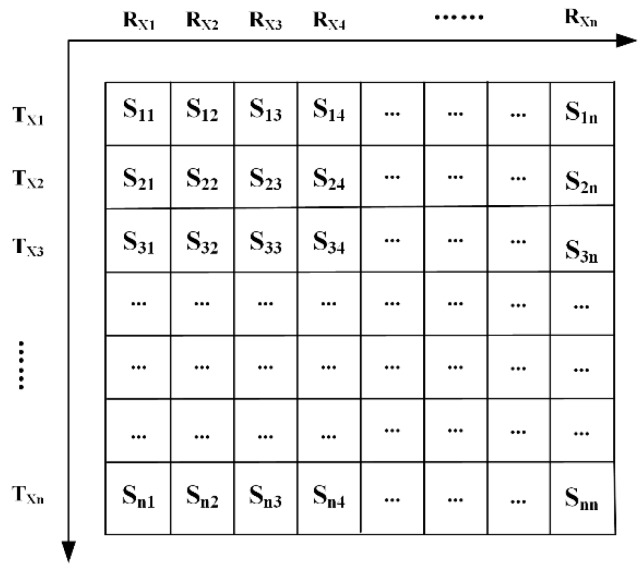
Full matrix data from ultrasonic phased array.

Based on its independent parallel reception channels, the ultrasonic phased array is powerful in data acquisition with full matrix capture (FMC). The dynamical focal depth and particular angle can be set by firing the array elements in a predefined sequence (often termed a delay law) online. Signals from all transmitter–receiver combinations can be captured. The time-domain data (A-scans) recorded are then automatically stored in the equipment, as displayed in Figure 1.

The symbols, *n*, *R*_X_, and *T*_X_, denote the total number of the array elements, transmitters, and receivers, respectively. For example, when *T*_X1_ is served as an excitation array element and all the array elements including *T*_X1_ are served as receivers, it generates *n* signals labeled as *S*_11_, *S*_12_, *S*_13_, …, *S*_1n_. Then, the remaining array elements are sequentially excited. Finally, *n^2^* response signals in total are generated. In this full matrix, the main diagonal signals are the self-transmitting and self-receiving signals from each array element. The whole procedure is similar to a B-scan in a ground-penetrating radar.

### 2.1. Diffuse Field in Rail

The research on ultrasonic diffuse waves deriving from room acoustics has been developed for many years. After a long time of propagation, there will be scattering and multiple reflections due to the wave–structure interaction, especially in a bounded material with complex geometry. Such a characteristic enhances the randomness of the elastic wave energy distribution. The long-lasting wavefield tends to be an equipartitioned wavefield after sufficient scattering, namely a diffuse field. In the irregularly shaped rails, the diffuse fields will also be generated. Very few works are attributed to the efficient detection and localization of defects in rails with ultrasonic diffuse fields.

Another important feature is that the Green’s function between two sensors can be retrieved by cross correlating the diffuse fields’ signal received by them. In practical experiments, it is important to generate a uniform diffuse field, which will directly affect the accuracy of the Green’s function reconstruction. The theory [10,28] also suggests that multiple reflections after a long enough time are helpful to the diffuse field signals’ extractions. The signal-to-noise ratio of the cross-correlation results gradually improve when the number of secondary sources or the duration of the diffuse fields increase.

**Figure 2:**
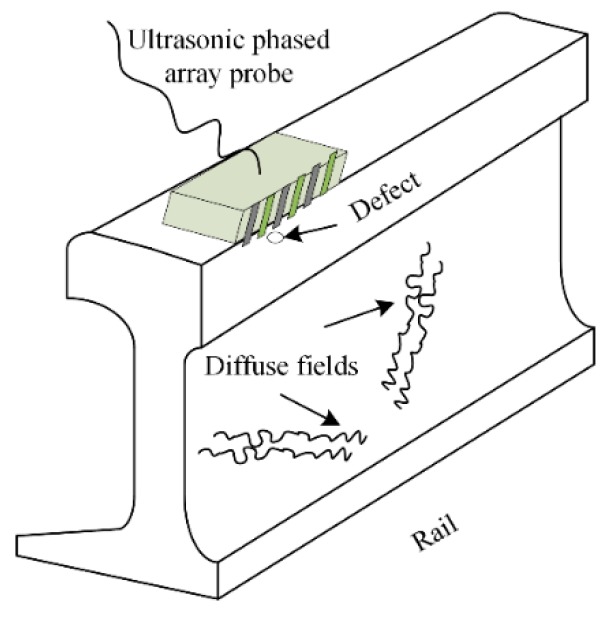
The experimental facility and diffuse fields in rail.

The methods of extracting the Green’s function are different in a conventional active and passive approach. The former uses a transmitting–receiving mode composed of a transmitter and a receiver to evaluate the Green’s function, while the latter only utilizes receivers to record ambient noise or diffuse field information. Ultrasonic phased array technology is analogous to the active approach. As mentioned above, the array elements are linearly distributed, which also leads to the nonuniform diffuse fields in the tested structure. However, fortunately, this does not weaken the theoretical basis for extracting the Green’s responses by using the diffuse fields. The schematic diagram of rail inspection is shown in Figure 2 with the phased array probe.

### 2.2. Full Matrix Reconstruction from Ultrasonic Phased Array

As analyzed in the previous section, the nonlinear saturation effect of the phased array probe makes the defect information in the early time, completely submerged under the condition of strong noise. The near-surface defects cannot be reconstructed directly from the full matrix, $h_{i,j}(t)$, which is directly obtained and named as the direct full matrix. Here, the symbols, *i* and *j*, correspond to a pair of transmitting and receiving elements’ number.

**Figure 3:**
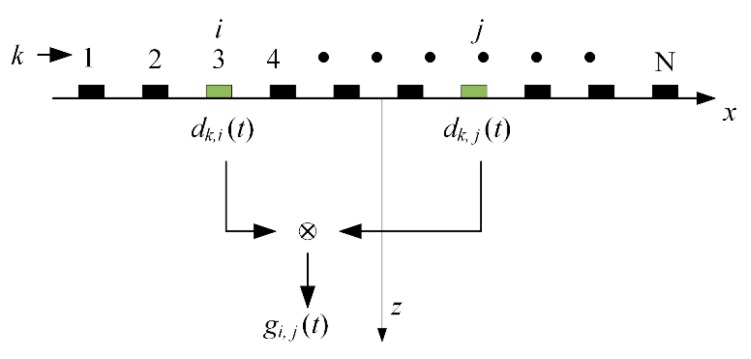
Green’s function retrieval using ultrasonic phased array.

In our experiments, the phased array was combined with the diffuse fields to extract the Green’s function, as shown in Figure 3. The Multi2000 system (M2M-NDT Co. Ltd., Les Ulis, France) was used to set and select the appropriate time-windowed signals in the diffuse fields. The starting time of the window is late enough, and the length of the window is large enough. With the characteristics of random noise fluctuation, the diffuse field is distributed throughout the rail. In this case, the full matrix, $d_{i,j}(t)$, can be obtained, which satisfies the characteristics of the diffuse fields, named as the diffuse full matrix. When the *k*th element is excited, the signals, $d_{k,i}(t)$ and $d_{k,j}(t)$, are acquired from the *i*th and *j*th receivers. Consequently, the cross-correlation, $C_{i,j}^{k}(t)$, between these receiving elements is:

(1) $$C_{i,j}^{k}(t)=\frac{1}{T}\int_{T_{c}+T}^{T}d_{k,i}(t)d_{k,j}(t+\tau )d\tau ,$$

where *T_c_* is the starting time of the recorded signals, and *T* is the duration of the time-windowed signals. In order to evaluate the Green’s function effectively, the remaining array elements were excited in turn. The Green’s function between two elements can be reproduced by an ensemble average of the cross-correlations, $C_{i,j}^{k}(t)$, which is an effective way [29]. The cross-correlation in the time domain between any two elements can be given by:

(2) $$C_{i,j}(t)=\frac{1}{n}\sum_{k=1}^{n}C_{i,j}^{k}(t).$$

Based on the time-derivative of the above equation, the reconstructed Green’s function full matrix was obtained:

(3) $$\frac{d}{dt}\left(C_{i,j}(t)\right)=g_{i,j}(t)\approx h_{i,j}(t).$$

To the best of our knowledge, the full matrix of the reconstructed Green’s function, $g_{i,j}(t)$, is approximately equal to the directly measured full matrix, $h_{i,j}(t)$. In most cases, the result, $C_{i,j}(t)$, contains unwanted redundancy, which ultimately leads to an unperfect symmetry of the Green’s function responses on the time axis. This reconstruction error has been called the cross-correlation redundancy elsewhere [30]. Therefore, in order to improve the final image resolution, it is necessary to improve the quality of the reconstructed full matrix, $g_{i,j}(t)$.

## 3. Experimental Signal Analysis

### 3.1. Experimental Setups

**Figure 4:**
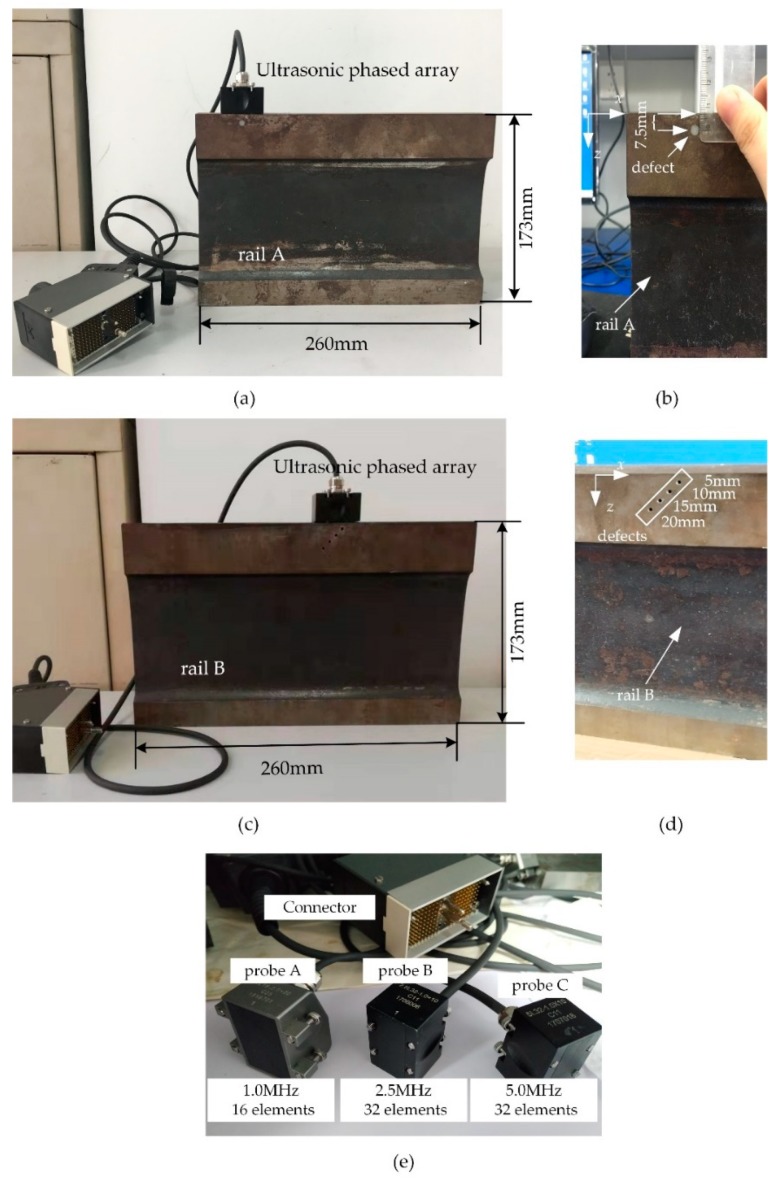
Rails and different phased array probes used in the experiment. (**a,c**) are diagram of the whole rail A and B respectively,(**b,d**) are the relative position information of the defects in rail A and B respectively, (**e**) is the different phased array probes.

Experiments were performed on rails, as shown in Figure 4a–d (corresponding to rails A and B). The height and length of the rails was 173 and 260 mm, respectively. A 5-mm diameter hole was drilled in rail A. The hole center was 7.5 mm to the surface of rail. Four holes with 2 mm in diameter were drilled and distributed on different depths labelled on rail B. In our experiment, the longitudinal wave velocity was 5900 m/s. Different ultrasonic phased array probes were used to investigate the imaging effects of the near-surface defects in rails as detailed in Figure 4e and Table 1. Unlike the case of guided waves [31], ultrasonic longitudinal waves in rail propagate along the z-axis direction. As long as the length of rails is much larger than that of transducer, the size of the rails does not affect the results. Additionally, according to the near field formula [32], $N_{d}=D^{2}/4\lambda$, where *D* is the transducer aperture and λ is the wavelength. Therefore, the defects are located in the near field because the distances of the defects from the rail surface (7.5 and 20 mm farthest from the surface) are less than $N_{d}$ of probe A, B, and C, which is about 42.85, 107.80, and 215.60 mm, respectively.

### 3.2. Signal Processing and Analysis

The information of the near surface can be obtained from the reconstructed full matrix, but the recovery of the reconstructed Green’s Function for the later-time signals is imperfect due to the limited number of statistical averages. Actually, the directly captured signals are more accurate than the reconstructed signal from the diffuse field for the later-time signals. Therefore, it is reasonable to combine the early information of the reconstructed signals with the later information of the signals directly captured. A hybrid full matrix, $f_{i,j}(t)$, with appropriate weights can be given [20]:

(4) $$f_{i,j}(t)=\frac{1}{1+e^{-\alpha (t-t_{c})}}h_{i.j}(t)+\beta (1-\frac{1}{1+e^{-\alpha (t-t_{c})}})g_{i,j}(t),$$

(5) $$\beta =\frac{\sum_{i=1}^{N}\left|h_{i,i}(t_{b})\right|}{\sum_{i=1}^{N}\left|g_{i,i}(t_{b})\right|},$$

where the parameter, β, is a compensation factor, which can be evaluated according to the arrival time, *t*_b_, of the first reflected echo signal; *t_c_* is the transitional time of the hybrid full matrix, and α is the smoothness of the transitional zone. The nonlinear saturation effect will be contained in the hybrid matrix if α is too small. On the contrary, artifacts will appear in the final image if α is too large.

**Figure 5:**
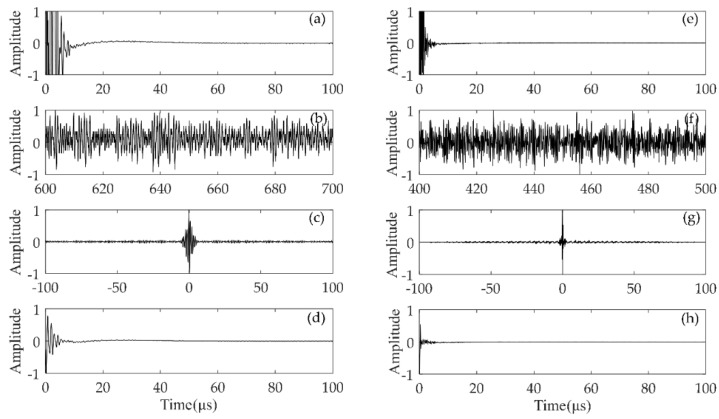
(**a**–**d**) are normalized signals for *i* = *j* = 8 corresponding to the full matrix, $h_{i,j}(t)$, $d_{i,j}(t)$, $g_{i,j}(t)$, and $f_{i,j}(t)$ with a 1.0 MHz excitation frequency and 16 elements. (**e**–**h**) are normalized signals for *i* = *j* = 16 corresponding to the full matrix, $h_{i,j}(t)$, $d_{i,j}(t)$, $g_{i,j}(t)$, and $f_{i,j}(t)$ with a 2.5 MHz excitation frequency and 32 elements.

In this subsection, we will mainly study the process of the diffuse full matrix, $d_{i,j}(t)$, and the hybrid full matrix, $f_{i,j}(t)$, to characterize the near-surface defect of rail A. Two types of probes, the one with 1.0 MHz excitation frequency and 16 elements, another with 2.5 MHz excitation frequency and 32 elements, were applied to the inspection of the near-surface defects in rails. Four different types of full matrix were obtained as shown in Figure 5. The time-domain signal was normalized to its maximum value. The results of $h_{i,j}(t)$ correspond to Figure 5a,e. It can be seen that the early time information is submerged by the blind zone caused by the ultrasonic phased array system. The matrix, $d_{i,j}(t)$, is the diffuse full matrix, which has random fluctuation as shown in Figure 5b,f. The matrix, $g_{i,j}(t)$, denotes the reconstructed Green’s function by using the cross-correlation of $d_{i,j}(t)$. Figure 5c,g denote the reciprocal Green’s function between two elements, so the Green’s function is an anti-symmetric function with respect to time. The full matrix, $f_{i,j}(t)$, is composed of $h_{i,j}(t)$ and the causal response of $g_{i,j}(t)$. As show in Figure 5d,h, it is suitable for near-surface defect imaging since the matrix, $g_{i,j}(t)$, contains the information of the near-surface zone and the matrix, $h_{i,j}(t)$, performs better in other areas of the rail.

## 4. Wavenumber Imaging

### 4.1. Theory for Wavenumber Algorithm

In this approach, the wave equation is used to solve the reflection interface of the internal defects in the workpiece. Firstly, the defects are discretized into some independent scatterers. Each of these scatterers is regarded as a secondary source, which generates an independent ultrasonic scattered field. Thus, the total scattered fields are the superposition of those independent scattered field. Finally, the specific position and size of the defects can be deduced according to the received signals of each array element. The whole process will be briefly discussed in the subsequent part of this subsection.

**Figure 6:**
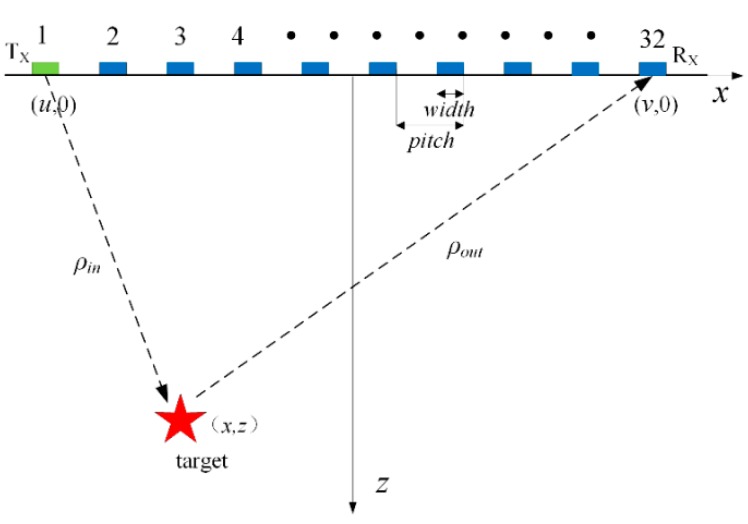
Geometry model of the transmitting and receiving process of ultrasonic phased array.

The full matrix wavenumber algorithm was applied to the ultrasonic field data. The 2-D geometry of ultrasonic phased array is displayed in Figure 6. In this figure, *X* and *Z* denote the lateral and depth directions. The scatterer is denoted by a star. The element width and adjacent elements spacing is represented by *width* and *pitch*, respectively, as shown in Figure 6. The coordinates of the transmitter and receiver are (u,0) and (v,0) respectively, so the distances from the target to the transmitter and receiver are $\rho_{in}=\sqrt{{\left(x-u\right)}^{2}+z^{2}}$ and $\rho_{out}=\sqrt{{\left(x-v\right)}^{2}+z^{2}}$.

In the frequency domain, a received signal can be expressed as:

(6) $$E(\upsilon ,u,\omega )=P(\omega )\iint f(x,z)g(\rho_{in},\omega )g(\rho_{out},\omega )dxdz,$$

where P(ω) is the spectrum of the transmitted signal and f(x,z) is the point spread function of the scatterer. The frequency-domain Green’s function responses from the transmitter and receiver to the scatterer are $g(\rho_{in},\omega )$ and $g(\rho_{out},\omega )$, respectively, which can be given by [33]:

(7) $$g(x,z,\omega )=\frac{-j}{4\pi }\int \frac{\exp(-j|z|\sqrt{k^{2}-k_{x}^{2}}+jk_{x}x)}{\sqrt{k^{2}-k_{x}^{2}}}dk_{x}.$$

Given that the wave velocity is denoted as *c* and the frequency is ω, the wavenumber can be expressed by k=ω/c. The spatial variables, *x* and *z*, correspond to the wavenumber variables, $k_{x}$ and $k_{z}$, in the 2-D space. Since ρ is given by *x* and *z*, g(ρ,ω) can be replaced by g(x,z,ω). Substituting Equation (7) into Equation (6), the received response is rewritten as follows:

$$E(\omega ,u,v)=P(\omega )\frac{-1}{{\left(4\pi \right)}^{2}}\iint \frac{\exp(jk_{u}u+jk_{v}v)}{\sqrt{k^{2}-k_{u}^{2}}\sqrt{k^{2}-k_{v}^{2}}}[\iint f(x,z)$$

(8) $$\times \exp(-j(k_{u}+k_{v})x-j(\sqrt{k^{2}-k_{u}^{2}}+\sqrt{k^{2}-k_{v}^{2}})z)dxdz]dk_{u}dk_{v},$$

where $k_{u}$ and $k_{v}$ are the wavenumber variables having correlations to spatial information of the transmitter (u,0) and the receiver (v,0), respectively. According to the relationship between the integral and the two-dimensional Fourier transform, Equation (8) is rewritten as:

(9) $$E(\omega ,k_{u},k_{v})=P(\omega )\frac{-F(k_{u}+k_{v},\sqrt{k^{2}-k_{u}^{2}}+\sqrt{k^{2}-k_{v}^{2}})}{{\left(4\pi \right)}^{2}\sqrt{k^{2}-k_{u}^{2}}\sqrt{k^{2}-k_{v}^{2}}},$$

where $F(k_{x},k_{z})$ is the spatial transform of the function, f(x,z). Keeping one of the parameters constant, e.g., the incident wavenumber, $k_{u}$, an inverse can be denoted by:

(10) $$\hat{F}(k_{x},k_{z}|k_{u})=-{\left(4\pi \right)}^{2}S^{-1}\{\sqrt{k^{2}-k_{u}^{2}}\sqrt{k^{2}-k_{v}^{2}}P^{*}(\omega )E(\omega ,k_{v}|k_{u})\}.$$

It is worth noting that there is a nonlinear conversion of coordinates in the above equation, which is the coordinate mapping ($S^{-1}\{\}$) from the data domain (corresponding to $k_{u}$, $k_{v}$, and k) to the image domain (corresponding to $k_{x}$, $k_{z}$). The above process, $S^{-1}\{\}$, is known as Stolt mapping [25] and is indispensable. That is because the distribution of data is evenly distributed in the data domain but not uniform in the image domain $\left(k_{x},k_{z}\right)$. In order to satisfy the condition of the inverse Fourier transform (the distribution of data is uniform), it is necessary to use a complex interpolation in the original frequency-wavenumber domain. The conversion relation is as follows [34]:

(11) $$k_{x}=k_{u}+k_{v},$$

(12) $$k_{z}=\sqrt{k^{2}-k_{u}^{2}}+\sqrt{k^{2}-k_{v}^{2}}.$$

After conversion and the inverse Fourier transform, the final image, $\hat{f}(x,z)$, is determined by:

(13) $$\hat{f}(x,z)=\frac{1}{{\left(2\pi \right)}^{2}}\iint \hat{F}(k_{x},k_{z})\exp(jk_{x}x+jk_{z}z)dk_{x}dk_{z}.$$

The kernel of the wavenumber imaging algorithm is operated by the Fourier transform, so it has the advantage of high-speed imaging. To sum up, the wavenumber algorithm combined with the full matrix data is summarized as the following steps: Firstly, a 3-D full matrix data is obtained, and 3-D Fourier transform is performed on this data; secondly, the 2-D data slice corresponding to each wavenumber, $k_{u}$, is mapped by Stolt complex interpolation, and the transformation of all data slices from $E(\omega ,k_{v}|k_{u})$ to $F(k_{x},k_{z}|k_{u})$ is implemented in turn; finally, $F(k_{x},k_{z}|k_{u})$ is added into $F(k_{x},k_{z})$ and the inverse Fourier transform is used to obtain the image of the target, $\hat{f}(x,z)$.

### 4.2. Near-Surface Defect Imaging in Rails

**Figure 7:**
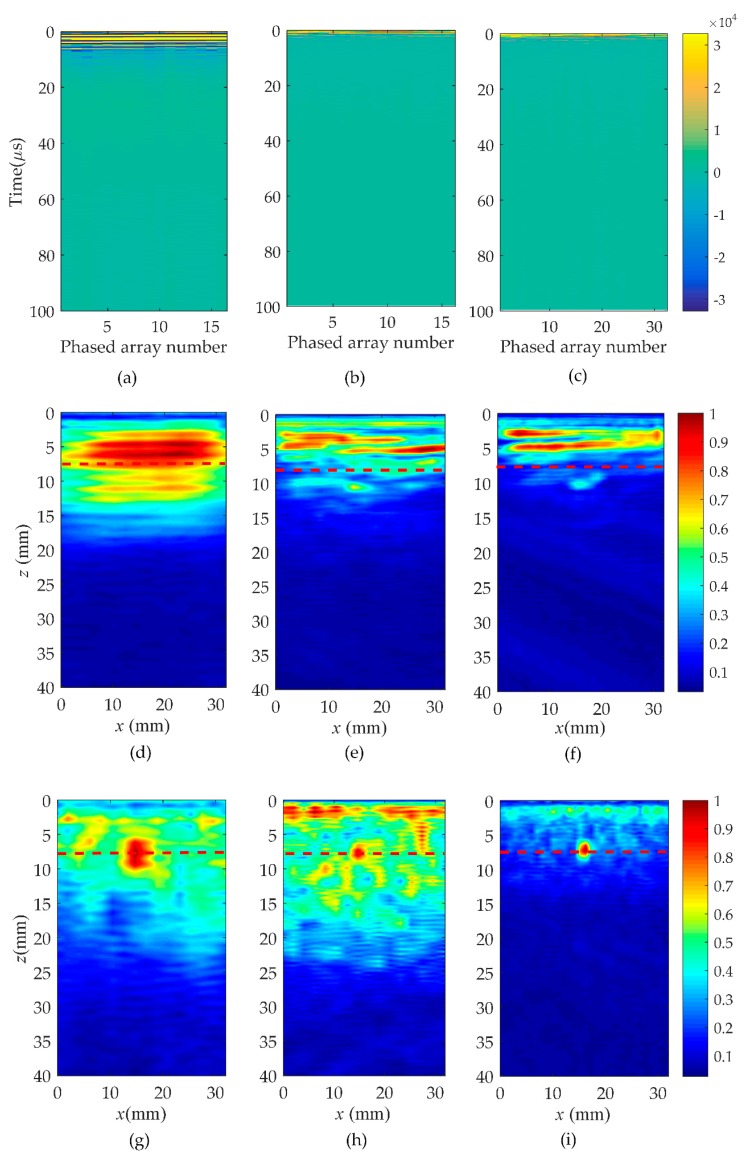
Comparison of B-scan and wavenumber imaging in three cases in rail A. Case one: (**a**) is pulse-echo responses, (**d**,**g**) are the images of the full matrix, $h_{i,j}(t)$ and $f_{i,j}(t)$; Case two: (**b**) is pulse-echo responses, (**e**,**h**) are the images of the full matrix, $h_{i,j}(t)$ and $f_{i,j}(t)$; Case three: (**c**) is pulse-echo responses, (**f**,**i**) are the images of the full matrix, $h_{i,j}(t)$ and $f_{i,j}(t)$.

**Figure 8:**
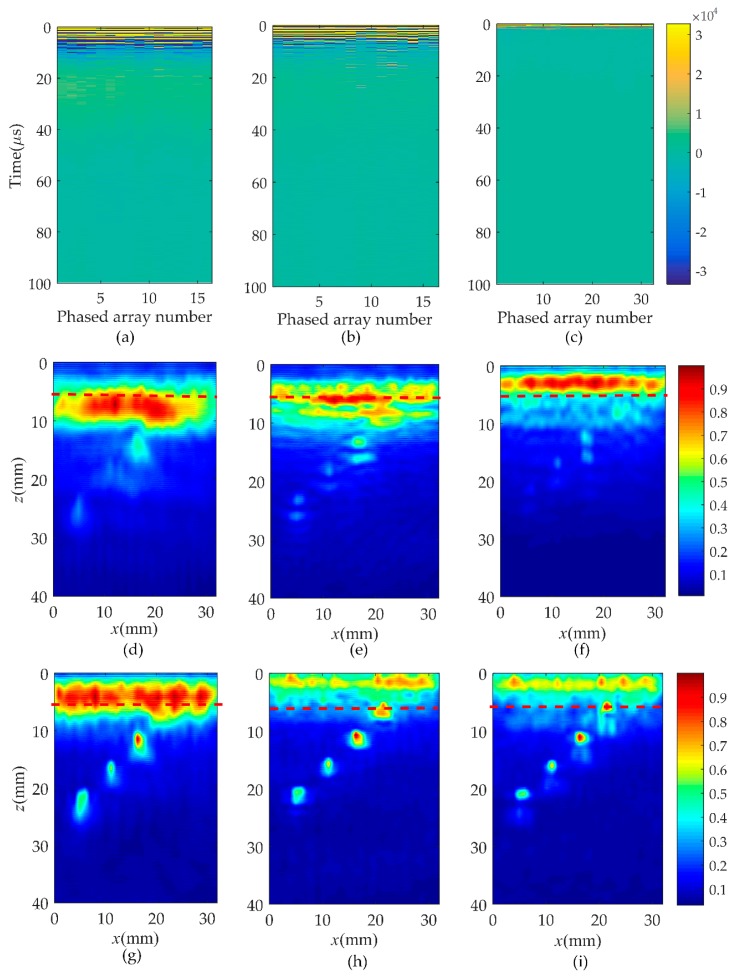
Comparison of B-scan and wavenumber imaging in three cases in rail B. Case one: (**a**) is pulse-echo responses, (**d**,**g**) are the images of the full matrix, $h_{i,j}(t)$ and $f_{i,j}(t)$; Case two: (**b**) is pulse-echo responses, (**e**,**h**) are the images of the full matrix, $h_{i,j}(t)$ and $f_{i,j}(t)$; Case three: (**c**) is pulse-echo responses, (**f**,**i**) are the images of the full matrix, $h_{i,j}(t)$ and $f_{i,j}(t)$.

In order to verify the validity of the hybrid full matrix ($f_{i,j}(t)$) for the localization of the near-surface defects, the wavenumber algorithm was applied in this subsection as shown in Figure 7 and Figure 8 (corresponding to rail A and rail B). The selection of different type of probes is listed in Table 2. Moreover, Figure 7a–c shows the pulse-echo responses, which is equivalent to a B scan for each element (*i* = *j*, the symbols *i* and *j* correspond to a pair of transmitting and receiving elements). Figure 7d–f are the wavenumber imaging results of the matrix, $h_{i,j}(t)$, corresponding to case one, case two, and case three. After the comparison of results, it was clearly found that the B-scans of all cases could not locate the location and size of defects. From the imaging results shown in Figure 7d–g, it can be noted that the region of the noise extends to about 20 mm in the *z* direction. This causes the defect located at 5 to 10 mm from the surface of the rail to be completely covered by surface noise. Although the depth of the blind zone decreases to 15 mm in case two and 10 mm in case three, it is still impossible to clearly localize the near-surface defect. However, from the results of Figure 7g–i, a defect whose position is marked by the red dashed line can be obviously observed. In agreement with the real location of the defect, the center of the reconstructed near-surface defect is located at the position of about 7.5 mm. Meanwhile, the experimental results show that the proposed method is no obstacle to multiple defects closer to the surface and smaller defects, as shown in Figure 8. It is impossible to get defect information from the pulse-echo responses in Figure 8a–c. The noise submerges the information of the near-surface defects in the wavenumber imaging results of the matrix, $h_{i,j}(t)$, but the defects can be detected due to a combination of the Green’s function retrieval theory and the wavenumber imaging algorithm. While changing the excitation frequency or the number of array elements, the defect still exists, and the location remains unchanged. Additionally, under the same element number, the higher the excitation frequency, the better the reconstructed defect image quality will be. Keeping the excitation frequency identical, the near-surface noise is gradually suppressed as the number of elements increases.

**Figure 9:**
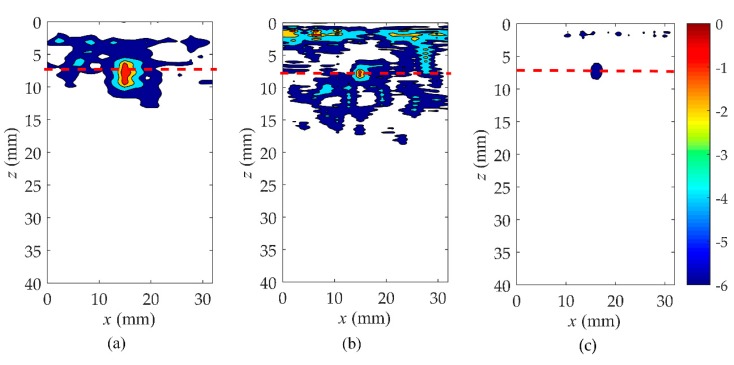
Comparison of point spread function in three cases in rail A. (**a**) Case one, (**b**) case two, (**c**) case three.

**Figure 10:**
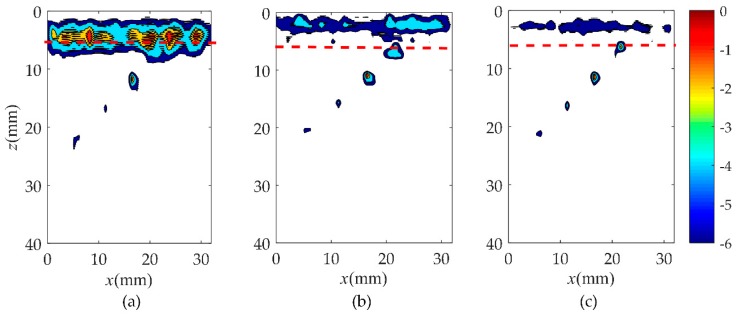
Comparison of point spread function in three cases in rail B. (**a**) Case one, (**b**) case two, (**c**) case three.

In order to further analyze the near-surface reconstruction of rail in these three cases, the results of the point spread function are presented. The point spread function is greater than–6 dB down from its maximum value, which is a common approach for expressing the imaging performance of an array [35]. According to the results of the three cases as shown in Figure 9 and Figure 10, the value of the point spread function in case three is the smallest and closest to the actual size. It is noticed that case one presented the worst imaging effect in all three cases, and the defect that is closest to the surface of rail B was still submerged by the noise. A higher excitation frequency and a greater number of elements could be attributed to a better defect image.

### 4.3. Influence of Diffuse Field Factors on Reconstructed Defects

In order to improve the quality of the reconstructed Green’s function and optimize the imaging of near-surface defects, further experiments were added in this section. The factors affecting the reconstruction include the number of elements, the duration, *T*, and the start time, *T_c_*, of the time-windowed signals. Among them, the influence of the number of elements was discussed above, so this section will mainly take the parameters, *T* and *T_c_*, into consideration. Rail A was selected as the workpiece and probe C was selected as the data acquisition device. The excitation frequency of the probe was 5.0 MHz and the sampling frequency was 25 MHz.

#### 4.3.1. The Duration, T, of the Time-Windowed Diffuse Field Signals

**Figure 11:**
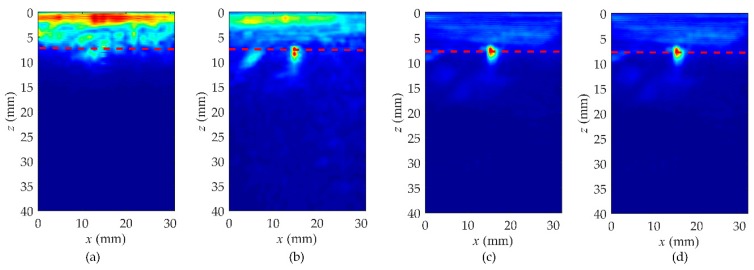
The study about the duration, *T*, of the time-windowed diffuse field signals in rail A. (**a**) *T* = 40 μs, (**b**) *T* = 80 μs, (**c**) *T* = 140 μs, (**d**) *T* = 160 μs.

As listed in Table 3, *T* was set as a variable and the other parameters remained constant to study the quality of the hybrid full matrix. Four different values of *T* were selected, with 40, 80, 140, and 160 μs. The imaging results are shown in Figure 11, and the position of the defect is also marked by the red dashed line. We can observe that the defect is mixed with the near-surface noise when *T* is equal to 40 μs. Although the defect can be roughly identified while *T* is equal to 80 μs, its shape is irregular. With the increase of *T*, the quality of the hybrid full matrix gets better and the imaging resolution is improved. When *T* reaches 140 μs, the near-surface noise is fully suppressed, and the shape and position of defect are all in accordance with the actual shape.

#### 4.3.2. The Starting Time, *T*_c_, of the Time-Windowed Diffuse Field Signals

**Figure 12:**
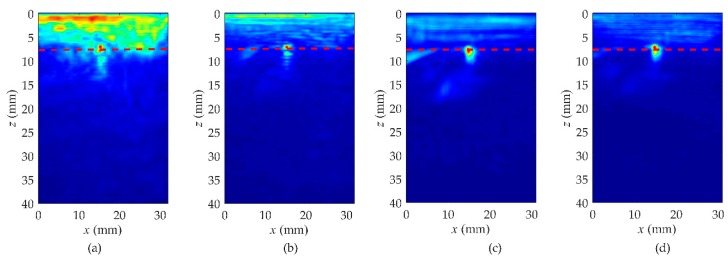
The study about the starting time, *T_c_*, of the time-windowed diffuse field signals in rail A. (**a**) *T_c_* = 200 μs, (**b**) *T_c_* = 300 μs, (**c**) *T_c_* = 400 μs, (**d**) *T_c_* = 500 μs.

Keeping the values of parameters *t_c_*, α, and *T* the same as detailed in Table 4, *T_c_* is a variable. The diffuse fields were obtained when *T_c_* was equal to 200, 300, 400, and 500 μs. From the imaging results of Figure 12, the defect is masked with the near-surface noise and cannot be distinctly separated from the near-surface noise when *T_c_* is 200 μs. However, with the increase of *T_c_*, the imaging quality is gradually improved.

In summary, the quality of the hybrid full matrix is directly associated with the number of elements in the phased array probe, the intercept time-windowed width, *T*, and the delay time, *T_c_*, of the diffuse field signals. The larger the size of the time window, *T*, is, the better the quality of the reconstructed Green’s function matrix will be. However, this also leads to a longer calculation time. If the *T_c_* value is too small, the characteristics of the diffuse field cannot be satisfied. Most importantly, after a large number of experiments, we also found that when the duration, *T*, is greater than 140 μs or the starting time, *T_c_*, is more than 400 μs, the imaging quality is not significantly improved. Therefore, the selection of the values of *T* and *T_c_* is close to 140 and 400 μs, respectively, for better reconstruction, which is reasonable in practical applications.

## 5. Conclusions

The diffuse fields in rails were first acquired by the ultrasonic phased array with FMC due to its angular inspection coverage and detection efficiency. For the detection of near-surface defects, the Green’s function response between the array elements was then reconstructed by the cross-correlation of their received signals. It was further weighted as a combination with the full matrix directly obtained. Finally, a wavenumber algorithm based on the Fourier-domain approach was used to realize the near-surface defects’ imaging. The major achievements of our paper are as follows:

1. The cross-correlation operation of the diffuse field signals realizes the reconstruction of the Green’s function between two elements. The reconstructed full matrix recovered the early time information of the near-surface defects submerged in the blind zone. Furthermore, a hybrid full matrix was used to get a better reflection of the interested area in rails.
2. The wavenumber algorithm was used to achieve the fast imaging using phased array probes with a different number of elements and excitation frequency. From the imaging results, it can be noticed that the imaging effects of near-surface defects have a relation with the number of elements and excitation frequency of the probes.
3. The influence of the duration, *T*, and the starting time, *T_c_*, of the time-windowed diffuse field signals on the reconstruction was discussed. The results indicate that with the larger window size and the longer starting time of the intercepted diffuse field signals, the imaging quality can be enhanced.

To some extent, this work provides a solution to realize defect detection in rails. It shows the great potential of Green’s function retrieval and wavenumber imaging to inspect near-surface defects in rails. The results presented here were preliminarily confined to defects in rails. Since the proposed method is essentially based on information processing and an imaging algorithm, it can be extended to anisotropic materials, such as the detection of fiber-reinforced composites, in the future.

## Figures and Tables

**Table 1:** The parameters of different phased array probes.

Phased Array Probe	The Number of Elements	Excitation Frequency	Element Width	Element Pitch
probe A	16	1.0 MHz	1.8 mm	2.0 mm
probe B	32	2.5 MHz	0.9 mm	1.0 mm
probe C	32	5.0 MHz	0.9 mm	1.0 mm

**Table 2:** The selection of different parameters of the probes.

Case	The Number of Element	Excitation Frequency
Case 1	16	1.0 MHz
Case 2	16	2.5 MHz
Case 3	32	2.5 MHz

**Table 3:** The parameters of the hybrid full matrix, $f_{i,j}(t)$.

Parameters	Value
*Tc*	500 μs
α	1.0 × 10^6^
*t_c_*	1.2 μs

**Table 4:** The parameters of the hybrid full matrix, $f_{i,j}(t)$.

Parameters	Value
*T*	120 μs
α	1.0 × 10^6^
*t_c_*	1.2 μs
